# Pharmacokinetics and pharmacodynamics utilizing unbound target tissue exposure as part of a disposition-based rationale for lead optimization of benzoxaboroles in the treatment of Stage 2 Human African Trypanosomiasis

**DOI:** 10.1017/S003118201300098X

**Published:** 2013-09-05

**Authors:** STEPHEN WRING, ERIC GAUKEL, BAKELA NARE, ROBERT JACOBS, BETH BEAUDET, TANA BOWLING, LUKE MERCER, CYRUS BACCHI, NIGEL YARLETT, RYAN RANDOLPH, ROBIN PARHAM, CINDY REWERTS, JACOB PLATNER, ROBERT DON

**Affiliations:** 1 SCYNEXIS Inc., Research Triangle Park, North Carolina, USA; 2 Haskins Laboratory, Pace University, New York, New York, USA; 3 Anacor Pharmaceuticals, Inc., Palo Alto, California, USA; 4 Drugs for Neglected Diseases Initiative, Geneva, Switzerland

**Keywords:** *In vitro* susceptibility, time-kill, pharmacokinetics–pharmacodynamics, tissue disposition, benzoxaboroles, HAT

## Abstract

This review presents a progression strategy for the discovery of new anti-parasitic drugs that uses *in vitro* susceptibility, time-kill and reversibility measures to define the therapeutically relevant exposure required in target tissues of animal infection models. The strategy is exemplified by the discovery of SCYX-7158 as a potential oral treatment for stage 2 (CNS) Human African Trypanosomiasis (HAT). A critique of current treatments for stage 2 HAT is included to provide context for the challenges of achieving target tissue disposition and the need for establishing pharmacokinetic–pharmacodynamic (PK–PD) measures early in the discovery paradigm. The strategy comprises 3 stages. Initially, compounds demonstrating promising *in vitro* activity and selectivity for the target organism over mammalian cells are advanced to *in vitro* metabolic stability, barrier permeability and tissue binding assays to establish that they will likely achieve and maintain therapeutic concentrations during in-life efficacy studies. Secondly, *in vitro* time-kill and reversibility kinetics are employed to correlate exposure (based on unbound concentrations) with *in vitro* activity, and to identify pharmacodynamic measures that would best predict efficacy. Lastly, this information is used to design dosing regimens for pivotal pharmacokinetic–pharmacodyamic studies in animal infection models.

## INTRODUCTION

Prediction of the therapeutic dose and success of an anti-parasitic agent requires an understanding of the complex interaction between the inherent potency of the agent, the clinically relevant exposure required within the host's target tissue and hence the organism, and the duration that exposure has to be sustained in that tissue to effect a cure. Potential disconnects between exposure in plasma and the target tissue must be considered early to ensure drug exposure is assessed in the pharmacologically relevant compartment (Louie *et al.* 2005). For example, in malaria, leishmaniasis, human African trypanosomiasis and African animal trypanosomiasis the relevant compartments would include blood erythrocytes, macrophages, brain and testes, respectively.

Determination of an effective dose for a given agent may be achieved empirically in pre-clinical studies by establishing a dose–response relationship; however, this approach is time consuming and requires extensive use of animal models, often comprising a large number of animals to overcome the variability inherent in these studies. Moreover, this approach may yield complete treatment failures because the candidate drug lacks the pharmacokinetic properties to achieve therapeutically relevant exposure even at the highest doses administered. Thus, for capacity, timeliness and ethical reasons establishing *in vivo* dose–response relationships, whilst acceptable for small numbers of well-characterized compounds, is often undesirable for comparison of larger numbers of compounds that maybe synthesized during the lead optimization phase of drug discovery programmes. Furthermore, extrapolation of the effective dose in a pre-clinical disease model to a clinically relevant dose required for humans may not be reliable if based on allometry alone.

**Fig. 1.**
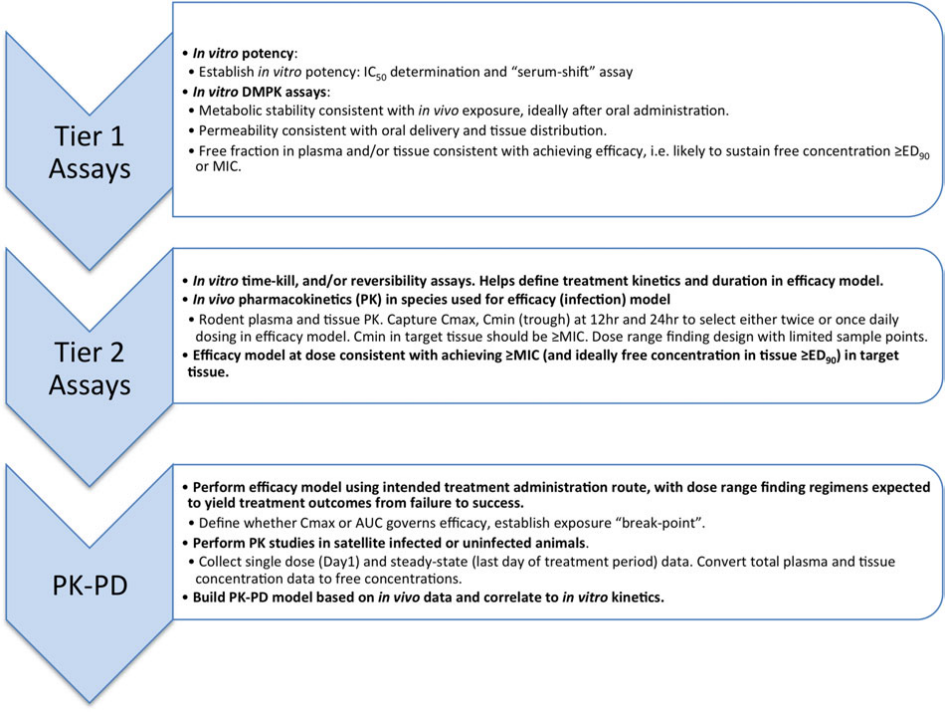
Progression pathway during lead optimization of benzoxaboroles as potential treatment for HAT.

**Fig. 2.**
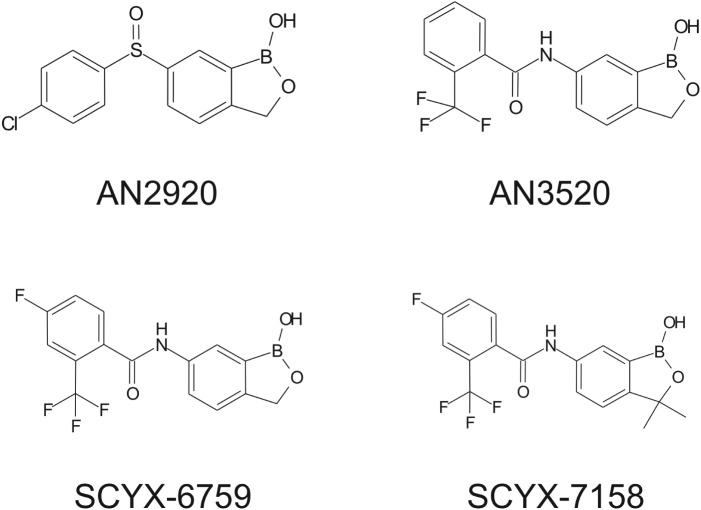
Structures of milestone benzoxaboroles.

In this review, we describe a data-driven strategy (Fig. 1) developed during lead optimization studies that led to the discovery and development of SCYX-7158 as a potential oral treatment for Human African Trypanosomiasis (HAT). The strategy employed target tissue disposition, complemented with *in vitro* susceptibility, time-kill and reversibility data to identify the pharmacokinetic–pharmacodynamic measures that were associated with efficacy in a murine model of stage 2 (CNS) HAT. Data are presented for milestone compounds evaluated during lead optimization (Fig. 2).

Human African Trypanosomiasis is caused by infection with the eukaryotic protozoan parasite *Trypanosoma brucei*, which is transmitted with the saliva through the bite of an infected tsetse fly. HAT currently affects thousands of individuals in sub-Saharan Africa (WHO, 2006). Complete clearance of the parasite from the body presents a key tissue distribution challenge for drug discovery efforts insomuch as it initially lives in the bloodstream (Stage 1 disease) of the infected individual, then later migrates across the blood–brain barrier (BBB) to establish the fatal, if untreated, central nervous system (CNS) infection (Stage 2). For complete cures it is important to maintain therapeutic drug levels in the haemolymphatic and CNS compartments to address both stages of the disease. The time between initial infection and CNS infiltration is highly variable, and dependent upon the strain of parasite (e.g. *Trypanosoma brucei gambiense vs Trypanosoma brucei rhodesiense*) (Masocha *et al.* 2007). Because the symptoms of HAT prior to CNS infiltration by the parasite are relatively mild and non-specific (e.g. transient fever, general malaise), and good diagnostic tests for the infection are not available, victims commonly progress to the CNS stage of the disease prior to seeking treatment.

Drugs to treat HAT, while available, are undesirable due to toxicity, difficulty of administration and ineffectiveness, particularly with respect to Stage 2 disease. The shortcomings of these treatments are discussed herein with emphasis on CNS disposition challenges and how they were addressed during the discovery of SCYX-7158. Data are compared for milestone compounds in the optimization campaign: AN2920, an early anti-trypansomal compound in the benzoxaborole series; AN3520, the first compound with oral pharmacokinetics commensurate with efficacy in the acute (Stage 1) murine HAT model, and SCYX-6759, the first compound to achieve 100% cures in the CNS (Stage 2) murine model following oral administration.

The optimization strategy employed *in vitro* time-kill and reversibility studies complemented with *in vivo* exposure data based on the total and unbound concentrations in plasma and target tissues to predict whether discovery-phase compounds had sufficient merit to progress to *in vivo* efficacy models, and if so, to design dosing regimens based on either single or multiple daily administrations that were most likely to provide sterile cures in rodent models. Taken together, we believe that the strategy, methods and data presented in this review provide a valuable starting point for other parasitic disease targets.

## MATERIALS AND METHODS

### Materials

Investigational compounds and the deuterium-labelled internal standard SCYX-5232 were synthesized by SCYNEXIS, Inc. (Research Triangle Park, NC) and were characterized by ^1^H-nmr and LC-MS before use. All compounds were purified to >95%. HPLC grade solvents were acquired from Fisher Scientific (Pittsburgh, PA). Formic acid (Fluka Analytical, ⩾98% purity) was purchased from Sigma-Aldrich (St. Louis, MO) and ammonium formate (Alfa Aesar, 99% purity) was purchased from VWR International, LLC (West Chester, PA). Phosphate buffered saline (PBS) was from Gibco (Invitrogen Corporation, Carlsbad, CA).

### Pharmacokinetic studies in mice

The CNS disposition and pharmacokinetics of the benzoxaboroles were evaluated in infected and uninfected mice following oral administration. *In vivo* phases of single-dose rodent PK studies were performed at Vivisource (Waltham, MA) following Institutional Animal Care and Use Committee (IACUC) review in an Association for Assessment and Accreditation of Laboratory Animal Care International (AAALAC)-approved facility. Studies in *Trypanosoma brucei brucei* (TREU 667 strain) infected mice, or uninfected satellite animals, were performed after IACUC review at Pace University (NewYork, NY).

Male CD-1 or female Swiss Webster mice (approximately 25 g) were administered test compounds by oral gavage either once (QD) or twice per day (Q12 h) with doses ranging between 6 and 100 mg kg^−1^ as described below. All compounds were formulated as an *in situ* sodium salt in 2% (*v/v*) ethanol, 5% (*m/v*) dextrose, to yield typically clear colourless solutions that were administered at 4 mL kg^−1^. Blood samples for pharmacokinetic studies were collected into polypropylene tubes containing K_2_EDTA anticoagulant and stored on wet ice until centrifuged and processed to plasma. Plasma was stored at −80 °C. Blood samples from mice were collected via cardiac puncture under terminal anaesthesia. Rodents were euthanized in a CO_2_ chamber before collection of terminal blood or tissue samples. Brain tissues were blotted dry and stored at approximately −80 °C.

### Analysis of test compounds in biological samples

#### Tissue homogenization

On the day of analysis either fresh tissue (for binding studies) or freshly thawed tissue samples from PK studies were blotted dry, weighed and homogenized in 1 volume of PBS. The density of brain tissue used for the determination of volume was 1·043 g mL^−1^ (Allen *et al.* 1959).

Whole tissue samples were homogenized in parallel by means of a Precellys^®^ 24 bead mill homogenizer (Bertin Technologies, France) using 1·5 mL Precellys tubes containing 1·4 mm diameter ceramic beads, (CK14), and a single 23 sec cycle of 6500 pulses per minute. Parallel bead homogenization increased throughput and avoided potential cross-contamination of samples as each was wholly contained and homogenized within its own sealed vial with no direct contact between the homogenizer instrument or other samples.

After homogenization, an additional volume of PBS was added to reduce viscosity. All tissue homogenates were treated in a bath sonicator at 22 °C for approximately 5 min to remove air bubbles.

#### Preparation of biological samples for LC-MSMS

Plasma (50 *μ*L) was treated with 4 volumes of ice-cold methanol (containing 25 ng mL^−1^ of either deuterium (d_6_)-labelled SCYX-7158 internal standard (SCYX-5232) for SCYX-7158 analyses or a related benzoxaborole for other compounds) to precipitate proteins. Treated samples were gently mixed on a 96-well plate shaker for approximately 10 min then centrifuged at approximately 3000 ***g*** and 15 °C for 15 min. Supernatants were transferred to fresh 96-well plates for analysis by means of LC-MSMS. Samples of tissue homogenate (50 *μ*L), or buffer from protein binding studies, were treated similarly to plasma samples.

Calibration standards and quality control samples were prepared in matched drug-free matrix prepared by combining homogenized tissues from control animals. Drug-free tissues and plasma were obtained from Bioreclamation, Inc. (Westbury, NY).

#### High-performance liquid chromatography with tandem mass spectrometry (LC-MSMS)

All biological samples were assayed by a verified LC-MSMS method employing two dimensional chromatographic separation to overcome ion suppression (Annesely, 2003) caused by co-elution of endogenous components with benzoxaboroles. In summary, treated samples were loaded onto Phenomenex (Torrance, CA) Synergi Polar RP, 4 *μ*, 50×2 mm extraction columns and subsequently back-eluted onto a Phenomenex Luna C8(2) 3 *μ*, 50×2 mm analytical columns protected by a Phenomenex C8 guard cartridge. Mobile phases for extraction and analytical columns consisted of 5 mm ammonium formate and 0·1% (v/v) formic acid in water (A), and 5 mm ammonium formate and 0·1% (v/v) formic acid in methanol (B). Samples were loaded onto the extraction column in 40% B and back-eluted onto the analytical column using a linear gradient to 50% B between 0·55 to 1·55 min after loading the sample. A linear gradient from 5 to 95% B over 4.5 min was employed for analytical chromatography. Test compounds eluted at approximately 3 min, and the total run time was 6 min.

The instrumentation for two dimensional LC-MSMS comprised two Agilent 1100 series pumps (Agilent Technologies Inc., Santa Clara, CA), interfaced to either a API-3000 or API-4000 triple quadrupole mass spectrometer (Applied Biosystems, Foster City, CA) via a turbo-ion electrospray source operated in positive ionization mode. Eluent flow between extraction and analytical columns and the mass spectrometers was directed using two Valco 10-port valves (Valco Instruments Company Inc., Houston, TX). Samples were introduced to the system via CTC Pal Autosampler (Leap Technologies, Carrboro, NC) set to inject 20 *μ*L aliquots of sample extract through a 10 *μ*L sample loop. The liquid chromatography system and all peripherals were controlled from within Analyst V.1.4.2 software.

Instrumental conditions for the mass spectrometer and precursor to product ion transitions were selected and optimized for sensitivity and selectivity. The MS/MS transitions for AN2920, AN3520, SCYX-6759 and SCYX-7158 were 292·2/133·2, 322·0/89·2, 340·1/191·1 and 368·0/191·0, respectively.

### *In vitro* prediction of BBB permeability and Pgp-mediated efflux transport

The propensity of test compounds to cross the BBB was examined using an *in vitro* MDCKII-hMDR1 monolayer assay (Polli *et al.* 2001). In summary, MDCKII-hMDR1 cells (Netherlands Cancer Institute, Amsterdam, the Netherlands) were seeded at a density of 3×10^5^ cells per well onto microporous polycarbonate membranes in 12 well Costar Transwell plates (Corning Inc., Lowell, MA). The cells were incubated for 3 days during which time they formed confluent monolayers. Trans-epithelial resistance (TEER) was measured for each insert to ensure the integrity of the monolayer (acceptable TEER >160 Ω cm^2^). The permeability and susceptibility for Pgp-mediated efflux was evaluated by adding each compound at a concentration of 3 *μ*m, in the presence or absence of 2 *μ*m GF120918 (a potent Pgp inhibitor), to the apical compartment. Competency of the Pgp efflux transporter was confirmed by assay of propranolol (non-substrate) and amprenavir (substrate). Cell monolayers were incubated in triplicate with shaking (160 rpm) at 37 °C in a 5% CO_2_-enriched humidified atmosphere for 1 h. Samples were removed from the apical and basolateral compartments after incubation and assayed for test compound concentrations by LC-MSMS. Values for mass balance, P_app_A−B, P_app_A−B+GF918, and absorption quotient (AQ) were calculated for each compound (Troutman and Thakker, 2003; Thiel-Demby *et al.* 2004). Acceptance criterion for mass balance was 70–120%.

### Plasma protein and tissue binding

Binding to plasma proteins, brain homogenates or proteins in serum-fortified HMI-9 media was determined by rapid equilibrium dialysis (RED) (Pierce, Rockford, IL) using a 48-well plate-based format according to the manufacturer's instructions. Briefly, test compound was added to fresh plasma (Bioreclamation, Liverpool, NY) at the required concentrations, freshly prepared brain homogenate or media. Duplicate aliquots of each sample were transferred into the sample chambers of the RED devices, and dialysis buffer (BupH PBS) was added to the buffer chambers. The plates were sealed and incubated at 37 °C for 4 h. After dialysis, samples collected from the buffer and tissue chambers were treated with ice-cold methanol (3 volumes for plasma or HMI-9 media, 4 volumes for brain) to precipitate proteins. The treated samples were centrifuged for 10 min at approximately 3000 ***g*** at 15 °C. The supernatants were assayed for test compound by LC-MSMS. Calibration standards and quality control samples were prepared in matched matrix and assayed with samples. Values for unbound and bound fractions and mass balance were calculated. Concordance of binding for each batch of plasma was confirmed by assay of warfarin, imipramine and carbamezapine. Acceptance criterion for mass balance was 70–120%.

### *In vitro* time-kill assays and reversibility of trypanocidal effects

The time and concentration dependency for oxaborole-mediated killing of *T. b. brucei*, under constant (time-kill) and transient (reversibility) drug-pressure, was determined as described previously (Nare *et al*. 2010).

In summary, time-kill kinetics were determined using the CellTiter Glo kit (Promega Inc., Madison, WI) to measure trypanosome ATP content as a real-time indicator of viability. Test compounds were evaluated over the concentration range 0·01–10·0 *μ*g mL^−1^ to provide concentrations from sub-therapeutic levels to >5 fold the predicted minimum inhibitory concentrations (MIC) or maximal effects. Test solutions were prepared in HMI-9 media and dispensed into white wall-clear bottom 96-well plates (Corning Inc., Lowell, MA). Trypanosomes (1×10^4^) were added to each well, incubated at 37 °C/5% CO_2_ and, at specified time intervals, CellTiter Glo reagent was added to lyse parasites. Plates were incubated for a further 10 min in the dark whereupon luminescence was determined using an EnVision Multilabel plate reader (Perkin Elmer, Waltham, MA). Time-kill parameters were determined from plots of parasite viability *vs* incubation time for each concentration tested. All incubations were performed in triplicate and data are presented as composite mean values.

Reversibility was assessed as the ability *T. b. brucei* parasites to recover from transient exposure (0–24 h) to test benzoxaboroles (0·02–10 *μ*g mL^−1^). Trypanosomes were seeded in clear 96-well ‘V’-bottom plates at a density of 1×10^5^ parasites per well; one plate was prepared for each time-point. After the designated incubation time, each plate was removed from the incubator and centrifuged at 4400 ***g*** for 5 min to sediment parasites. The supernatant containing the test compound was aspirated and three washing cycles were performed where 100 *μ*l of warmed HMI-9 media was added to each well and then aspirated. After washing, the parasites were re-suspended in 100 *μ*l of warmed media and 3×20 *μ*l aliquots of each suspension were transferred to fresh plates containing 80 *μ*l of fresh HMI-9 media per well. Thus, each concentration and time-point was evaluated in triplicate. Each plate was incubated for a further 72 h, whereupon, trypanocidal activity was determined using resazurin (Sigma-Aldrich, St. Louis, MO) where 20 *μ*l of a 25 mg/100 mL stock in PBS was added, incubated for an additional 4–6 h and fluorescence measured using the EnVision plate reader (excitation wavelength, 530 nm and emission wavelength, 590 nm).

### The effects of serum proteins on trypanocidal activity

To assess the potential effects of protein binding on trypanocidal activity, parasite viability assays were performed in the presence of increasing concentrations (2·5–50%) of foetal calf serum (Invitrogen, Carlsbad, CA) in HMI-9 media. *T. b. brucei* parasites were conditioned to the test serum concentrations for at least one round of passage prior to assay. Test compounds were added, incubated for 72 h, signal measured and IC_50_ determinations were conducted as described above using the resazurin endpoint. The fold change in IC_50_ from minimal serum concentration to maximal serum concentration was calculated and used to compare compounds and their potential for being restrictively protein bound.

### Efficacy in the murine model for chronic (Stage2, CNS) HAT

The efficacy of benzoxaboroles was evaluated in a murine model of Stage 2 (CNS) HAT (Nare *et al.* 2010; Jacobs *et al.* 2011). In summary, mice (*n* = 10 per group) were infected by bolus i.p. injection of 1×10^4^ *T. b. brucei* (TREU 667) parasites. A single bolus i.p. dose (10 mg kg^−1^) of berenil (Sigma-Aldrich, St. Louis, MO) was administered on day 4 as a positive treatment control for haemolymphatic-stage infection. Infection of the remaining animals proceeded for 21 days to establish a CNS infection before treatment with either a single dose of berenil (as above) or test compounds by the oral route either once or twice daily over 7 days at the doses indicated below. Animals were checked at least weekly for parasitaemia and were immediately removed from cages and euthanized upon recrudescence. Animals were considered cured if they were aparasitaemic for at least 180 days after treatment.

## RESULTS AND DISCUSSION

Until recently, progress towards new treatments for HAT had been slow, owing to a lack of commercial interest and, most notably, a poor understanding of CNS disposition and pharmacokinetic–pharmacodynamic (PK–PD) relationships for Stage 2 drugs in particular. This shortcoming is currently being addressed by academic and non-profit drug research and development (R&D) organizations including the Drugs for Neglected Diseases *initiative* (DND*i*), whose success has been demonstrated by delivery of two HAT compounds, Fexinidazole (Torreele *et al.* 2010) and SCYX-7158 (Jacobs *et al.* 2011), into clinical development. The target product profile (TPP) for these new agents included the requirement for oral delivery and the ability to treat both stages of the disease using a single dosing regimen. The PK–PD strategy described herein reflects the approach developed for the optimization and selection of SCYX-7158 as a pre-clinical and clinical candidate for the treatment of Stage 1 and Stage 2 HAT.

### Pharmacokinetic–pharmacodynamic measures

**Fig. 3.**
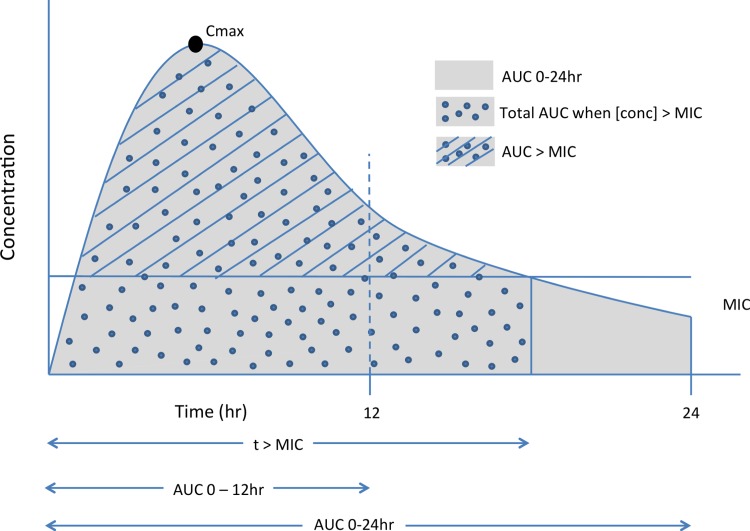
Representation of the potential pharmacokinetic–pharmacodynamic measures for modeling.

Pharmacokinetic–pharmacodynamic (PK–PD) relationships establish whether a drug's efficacy increases with dose and, if so, help determine which PK parameter can best be used with MIC to predict outcome. Typical measures used to predict efficacy are summarized in Fig. 3 and include: (a) the time above MIC (*t*>MIC), (b) the ratio of peak concentration and MIC (*C*_max_/MIC), (c) the ratio of the area under the concentration *vs* time profile (AUC) while concentration is greater than MIC, or (d) the ratio of the area under the concentration *vs* time profile (AUC) measured over the dosing interval or 24 h to MIC (AUC_0−*t*_/MIC) (Mueller *et al.* 2004). The most predictive measure guides whether an optimized dosing regimen employs either a fewer number of larger doses where efficacy is driven by *C*_max_, or more frequent but lower doses to maintain an AUC/MIC (average concentration/MIC) multiple. In either regimen it is generally desirable to maintain tissue concentrations above the MIC throughout the treatment period to lower the risk of the target organism generating resistance.

### Target tissue disposition for HAT

#### CNS disposition challenges for currently approved Stage 2 HAT drugs

One current first line treatment for Stage 2 HAT, melarsoprol, has been shown to be highly toxic, causing drug-induced death in 2–10% of patients (Schmid *et al.* 2004; Doua *et al.* 2005; Kuepfer *et al.* 2012). Nonetheless, it is active in treating both *T. b. gambiense* and *T. b. rhodesiense*. Reports of treatment failures with melarsoprol (Matovu *et al.* 2001) are suggestive of development of drug resistance (Delespaux and de Koning, 2007) although evidence of significant change in the sensitivity of *T. b. gambiense* strains to melarsoprol appears elusive (Likefack *et al.* 2006). Another possibility is that treatment failures may arise from inconsistent or inadequate CNS exposure of this drug. Indeed, failure to achieve sustained cerebrospinal fluid (CSF) exposure (a surrogate for the unbound fraction in brain tissue (Shen *et al.* 2004; Summerfield and Jeffrey, 2006; Lin, 2008)) is thought to contribute to the high (3–30%) failure rate with melarsoprol treatment (Legros *et al.* 1999). Pre-clinical and clinical studies support this claim. Following intravenous administration (doses of 2·2–3·6 mg kg^−1^) maximum concentrations of melarsoprol in the CSF of uninfected vervet monkeys were very low (∼50 ng mL^−1^) despite serum levels of the drug being substantially higher (1·7–3·8 *μ*g mL^−1^) (Burri *et al.* 1994). Equivalent concentrations of melarsoprol have been reported in both the serum (0·87–2·2 *μ*g mL^−1^) and CSF (39–79 ng mL^−1^) of human patients 24 h after the last injection of a multi-day treatment regimen (Burri and Keiser, 2001). The *in vitro* MIC reported for melarsoprol is 2–100 ng mL^−1^ (Burri *et al.* 1994) suggesting therapeutic levels may neither be achieved nor sustained. Increasing dose is contra-indicated because of a higher risk of melarsoprol-induced encephalopathy.

An alternative treatment for Stage 2 HAT, eflornithine, while better tolerated than melarsoprol, is potentially less attractive because it is active only against *T. b. gambiense* and must be administered by large intravenous infusion doses (400 mg kg^−1^ day^−1^) four times per day over fourteen days (Priotto *et al*. 2008). This treatment paradigm is very difficult to achieve in disease-endemic regions. Recently, clinical studies have shown that treatment duration maybe shortened to 7 days using a combination therapy of nifurtimox and twice daily eflornithine (Priotto *et al.* 2007); however, the complexity of parenteral administration remains.

Like melarsoprol, CNS exposure of eflornithine is limited. In mice, maximum concentrations of eflornithine only reach 10–40 nm, representing <0·1% of the administered eflornithine dose, and far below the reported *in vitro* MIC for the drug (ca. 50 *μ*m) (Sanderson *et al.* 2008). In this study, the authors included uninfected mice as controls to allow comparison of CNS exposure in healthy animals to those infected with *T. b. brucei* GVR35, a strain of the parasite commonly used in murine models of Stage 2 HAT (Jennings and Gray, 1983) as it readily establishes a CNS infection. The permeability of eflornithine across the BBB was similar between infected and uninfected animals for 21 days following infection, although greater permeability was observed for late-stage infected mice at days 28 and 35 post-infection. Sanderson *et al.* (2009) have also reported that the integrity of the BBB is maintained until late stage CNS infection. These observations, coupled with reports of interactions of *T. brucei* with the BBB in both *in vitro* (Grab *et al.* 2004) and *in vivo* (Masocha *et al.* 2007) models suggest a dynamic translocation of parasites between the periphery and the brain, with only modest modification of the BBB. Consequently, stage 2 drugs must be able to circumvent the physical and biological barriers present in the fully intact BBB to the extent that they achieve and maintain therapeutic levels within brain parenchyma and CSF. Reports for the pharmacokinetics of eflornithine in human patients are sparse but are consistent with those derived from animal models, suggesting that CSF levels of eflornithine at steady state are generally low (5–150 nm) relative to concentrations required to be efficacious *in vitro* (Milford *et al*. 1993).

### Progression pathway and identification of pharmacokinetic–pharmacodynamic measures based on unbound target tissue exposure

A data-driven strategy (Fig. 1) was developed during lead optimization studies for SCYX-7158 that has broad applicability in drug discovery for parasitic disease targets. The strategy employs ‘Tier 1’ *in vitro* metabolism and disposition assays, with *in vitro* parasite susceptibility assays, to prevent progression of compounds that would likely fail in animal infection models. Tier 1 assays may also prioritize compounds for ‘Tier 2’ *in vivo* target tissue disposition, and *in vitro* time-kill and reversibility assays used to identify the pharmacokinetic–pharmacodynamic measures that may be associated with efficacy in animal infection models such as the murine model of Stage 2 (CNS) HAT. *In vitro* and *in vivo* PK–PD measures may then be correlated to provide a rationale for clinical dosing regimens.

### Tier 1: goals for potency and drug-like properties during lead optimization

#### *In vitro* susceptibility of *T. brucei*

Demonstration of *in vitro* potency and selectivity against a target organism *vs* host is typically the first hurdle in advancing a compound through the progression pathway to pre-clinical candidate selection. The milestone oxaborole carboxamides (Fig. 2, Table 1) each exhibited potent activity against *T. b. brucei, T. b. rhodesiense and T. b. gambiense in vitro*, and achieved >40 fold selective inhibition of parasite compared to the L929 mammalian cell line employed as an *in vitro* predictor of cytotoxicity and therefore selectivity (Nare *et al.* 2010). The IC_50_ is defined as the concentration of compound that is required to decrease parasite viability by 50%, and the minimum inhibitory concentration (MIC) is the lowest concentration that completely inhibits parasite growth (Jacobs *et al.* 2011).

**Table 1.** *In vitro* activity of milestone benzoxaboroles against *T. brucei* subspecies

Compound	IC50 (*μ*g mL^−1^)									Selectivity
	*T. b. brucei*	*T. b. rhodesiense*	*T. b. gambiense*							
	427	STIB900	108R	40R	DRANI	DAL 1402	ITMAP 141267	STIB930	L929	
AN2920	0·17	0·13	0·1	0·11	0·094	0·046	0·06	NA	8·36	*⩾*49
AN3520	0·04	0·04	NA	NA	NA	NA	NA	0·01	3·77	*⩾*94
SCYX-6759	0·07	0·04	0·03	0·04	0·038	0·017	0·024	0·03	9·51	*⩾*136
SCYX-7158	0·29	0·29	0·17	0·36	0·129	0·065	0·092	NA	50	*⩾*138

#### *In vitro* DMPK assays: establishing drug-like properties

Compounds with *in vitro* anti-parasitic activity must also demonstrate adequate ‘drug-like’ properties; namely, solubility, *in vitro* metabolic stability in the presence of liver sub-cellular fractions (typically microsomes or S9 fraction) (Mandagere *et al.* 2002), non-restrictive binding to plasma proteins (Mackichan, 2005; Summerfield *et al.* 2006) and, for oral compounds, permeability in either MDCKII-hMDR1 or Caco-2 monolayer assays (Mandagere *et al.* 2002). The latter can also provide insight on the compound's ability to cross blood-tissue barriers such as the blood–brain, blood-testicular or blood-retinal barriers, and interact with drug efflux or uptake transporters that can either attenuate or enhance disposition within a target tissue.

The ability of a drug to cross the BBB and gain entry into the CNS compartment reflects interplay between membrane permeability, protein binding and drug transporters, notably efflux transporters such as P-glycoprotein (Pgp) or Breast Cancer Related Protein (BCRP). It is also worth considering that these parameters may impact the ability of a compound to penetrate the target parasite.

In a study comparing the properties of CNS and non-CNS antihistamines, compounds with high brain-to-plasma concentration ratios typically achieved high passive permeability (>150 nm s^−1^) in MDCK-MDR1 transport assays, moderate protein binding (<84%), and were not Pgp substrates (Mahar Doan *et al.* 2004). In contrast, compounds with low brain-to-plasma ratios typically displayed low passive permeability (<100 nm s^−1^), were substrates for Pgp and had higher binding to plasma proteins. Exceptions, such as terfenidine, were noted which, although being Pgp substrates, had high passive permeability that was able to overcome the transporter-mediated efflux (Polli *et al.* 2001). *In vitro* permeability, although a helpful predictor of CNS penetration, reflects the potential rate and is a surrogate for the actual extent of brain drug delivery. The latter is the more helpful parameter for developing models and data on unbound tissue exposure are preferred for developing PK–PD models.

**Fig. 4.**
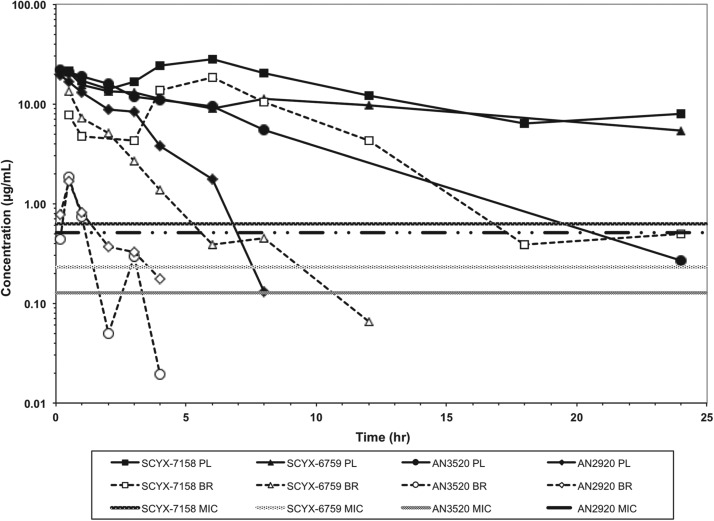
Total concentration *vs* time curves for AN2920, AN3520, SCYX-6759 and SCYX-7158 in male CD-1 mice following a single oral dose. Data are normalized to a 50 mg kg^−1^ dose to allow direct comparison of exposure between compounds. The MIC line is defined as the lowest concentration of each compound that completely inhibits visible parasite growth. Data points for plasma (solid lines) and brain (dotted lines) represent a single mouse at each time point.

*In vitro* protein binding and permeability data for the milestone benzoxaboroles during the discovery of SCYX-7158 are presented in Table 2. Oxaboroles as a class generally met the criteria for CNS penetration with milestone compounds demonstrating very high permeability values (P_appA–B_, >350 nm s^−1^) in the MDCK-MDR1 transport assay, and were not substrates for the Pgp efflux transporter (AQ<0·3 (Thiel-Demby *et al.* 2004)). Less desirably, protein binding was modest to high 93·2–98·7% and likely hampered CNS penetration such that in single-dose studies in mice the brain to plasma ratio was typically less than unity (Fig. 4). Protein binding was also a concern because it may also attenuate *in vivo* potency.

**Table 2.** Comparison of *in vitro* protein binding and MDCKII-hMDR1 permeability

	*In vitro* study	Compound			
		AN2920	AN3520	SCYX-6759	SCYX-7158
Binding to plasma protein	Human plasma (1 *μ*g mL^−1^, *n*=3)				
	Fraction bound (%)	93·2±0·836	95·3±0·425	97·7±0·053	98·7±0·018
	Unbound fraction (%)	6·8	4·7	2·3	1·3
	Mass balance (%)	106	110	97	110
MDCK-MDR1 Permeability	Incubation concentration (*μ*m)	3	3	3	3
	Papp, A–B direction (nm s^−1^)	438	424	379	414·8
	Papp, A–B direction+GF918 (nm s^−1^)	465	434	386	426·9
	Mass balance (A–B, A–B+GF918, %)	94%, 99%	92%, 90%	89%, 95%	79%, 75%
	Absorption quotient	0·06	0·02	0·02	0·03
	P-gp classification	Non-substrate	Non-substrate	Non-substrate	Non-substrate

#### Serum shift assay and the impact of drug binding to plasma proteins or tissues on potency

**Fig. 5.**
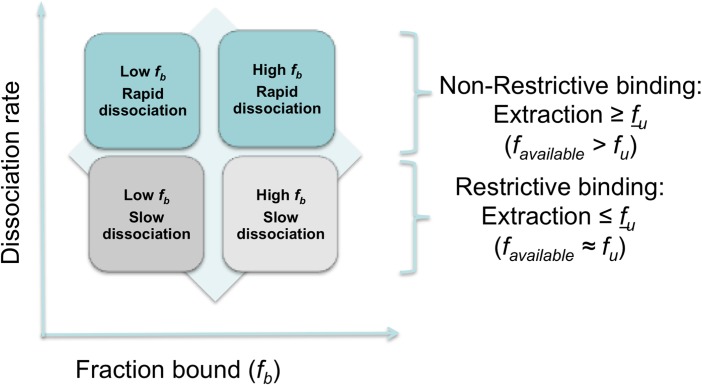
Representation of restrictive and non-restrictive binding.

**Fig. 6.**
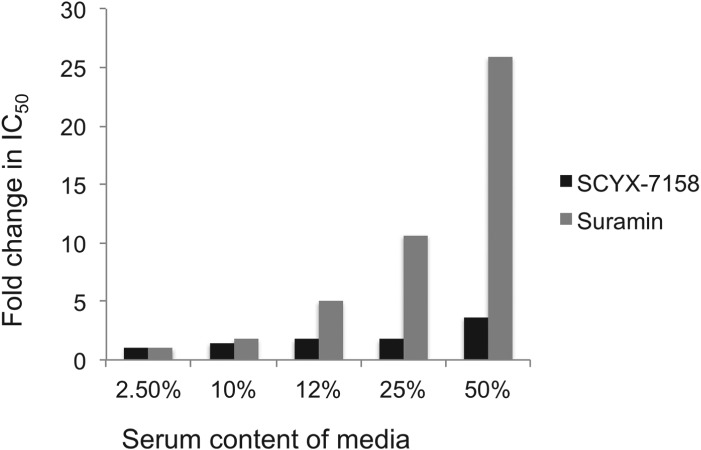
Impact of serum on the *in vitro* potency of SCYX-7158 and suramin as determined by means of the serum-shift assay. Data represent fold change in IC_50_ against *T. b. brucei* relative to the lowest serum concentration evaluated (2·5%). IC_50_ values were determined from composite mean values for triplicate assays at each concentration of either SCYX-7158 or suramin.

Drug distribution in plasma and tissue reflects equilibrium between bound and unbound (free) fractions, where the unbound fraction is deemed to be pharmacologically active and also the target for drug clearance mechanisms. As unbound drug is cleared from a compartment either by elimination mechanisms or, for example, uptake into a target cell or parasite, bound drug in plasma or tissue interstitial spaces dissociates to re-establish the equilibrium. Restrictive or non-restrictive binding has been used qualitatively to describe the dissociation rate (*K*_*d*_) that, rather than the binding extent, influences whether binding impacts potency (Fig. 5) (MacKichan, 2005). Despite its importance, *K*_*d*_ is rarely measured during lead optimization of ‘small molecule’ drugs owing to the complexity of the measurement. More frequently, the *in vitro* ‘serum-shift’ assay is performed as a surrogate that assesses the impact of increasing serum concentration on potency (IC_50_). In this assay, the trypanocidal activity of benzoxaboroles appeared only modestly attenuated by serum. In a comparison, the fold changes in IC_50_ between 2·5 and 50% calf serum for SCYX-7158 and suramin were 3·6 and 26, respectively, suggesting SCYX-7158 is less restrictively bound (Fig. 6). A marked loss in potency in a serum-shift assay provides a warning that *in vitro* potency in a low serum media may not translate to *in vivo* efficacy where the candidate drug maybe bound thereby achieving sub-therapeutic concentrations. As a general strategy it is more reliable to build PK–PD models based on unbound concentration in the target compartment rather than total drug levels.

#### Unbound fraction and concentrations in target tissues

CSF has been employed successfully as a surrogate for the unbound fraction in brain tissue. However, systemic drugs can enter the CSF by 2 main routes; either indirectly via passage across the BBB followed by diffusion from the brain interstitial fluid into the CSF, or directly via passage across the choroid plexus. In the latter instance, drug in CSF would thus not represent unbound concentration in brain interstitial fluid. A further complication of using CSF is the practical challenge of collecting an adequate volume from mice that is also free of contamination by blood. Consequently, *in vitro* ultrafiltration or equilibrium dialysis techniques, with either fortified samples of tissue homogenate or *ex vivo* tissue samples (Fridén *et al.* 2007; Watson *et al.* 2009), are most practicable for assessing unbound concentrations in tissues.

**Fig. 7.**
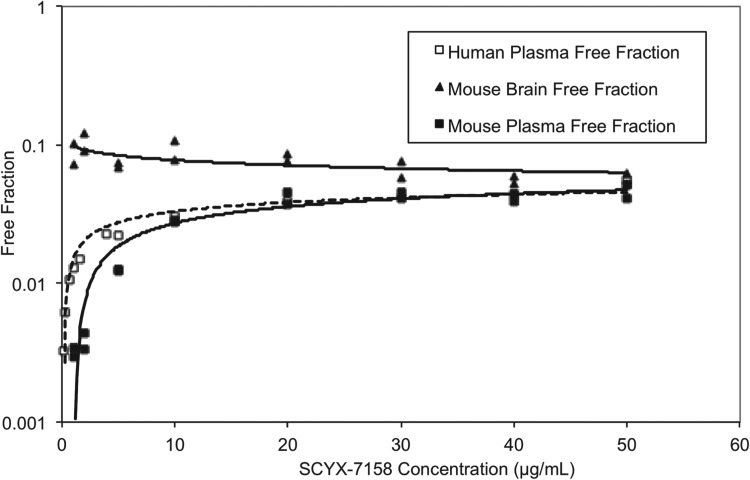
The concentration dependent *in vitro* binding of SCYX-7158 to mouse and human plasma, and mouse brain tissue determined by equilibrium dialysis. Binding studies were performed in fresh tissues, each data point represents the mean of at least triplicate measurements.

At steady state, permeable compounds unaffected by active uptake or efflux mechanisms should achieve generally similar unbound concentrations in tissue and plasma although the unbound fractions may differ. The binding of SCYX-7158 to mouse and human plasma was concentration dependent, with the unbound fraction increasing from 0·3 to 4·6% and from 0·3 to 3·9% in mouse and human plasma, respectively (Fig. 7). The most rapid increase in the unbound fraction occurred between 0·1 and 10 *μ*g mL^−1^ SCYX-7158. Interestingly, the binding to mouse brain tissue was generally similar across the concentration-range studied (Fig. 7). The relationship between unbound fraction in each matrix and total concentration was calculated to allow conversion from total to free concentrations in PK studies. The SCYX-7158 concentration dependent binding to proteins in the cell culture media used for *in vitro* trypanosomal assays was also determined to allow analysis of time-kill and reversibility data, based on free concentrations and to identify the IC_50u_ and MIC_u_ based on free concentration.

To progress to Tier 2 assays, a compound should be non-cytotoxic to mammalian cells and suitable for administration by a route and dose likely to achieve and ideally maintain ⩾ED_90_ unbound concentration in plasma or tissue.

### Tier 2: goals for tissue disposition and efficacy in infection models

#### *In vitro* time-kill and reversibility of exposure to benzoxaboroles

Time kill and reversibility curves based on total drug exposure for the milestone benzoxaboroles, including SCYX-7158, have been reported previously (Nare *et al.* 2010; Jacobs *et al.* 2011). In summary, time-kill studies demonstrated that the onset of trypanocidal behaviour was rapid (<24 h) and concentration dependent, reaching maximal effect at approximately 4 fold the MIC. Transient exposure to benzoxaboroles in reversibility studies further demonstrated that a persistent effect on trypanosome survival was dependent on both exposure time and concentration.

**Fig. 8.**
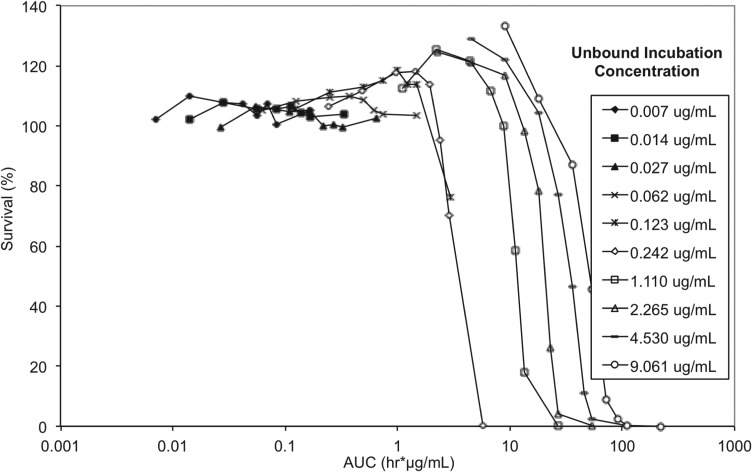
*In vitro* reversibility plots with presenting survival of *T. b. brucei vs* cumulative AUC based on unbound concentration. Experiments were conducted in triplicate and represent survival of treated parasites expressed as a percentage relative to untreated parasites.

To elucidate whether either drug concentration, AUC/MIC or exposure time have the greater impact on trypanosome survival, and to allow correlation between *in vitro* and *in vivo* efficacy endpoints, exposure time and concentration in the reversibility assay were considered in the context of exposure expressed as AUC (area under the concentration *vs* time curve) for unbound concentrations (Fig. 8).

The lowest AUC affording a 100% trypanocidal effect (5·81 *μ*g h mL^−1^) was achieved following incubation with an unbound concentration of 0·242 *μ*g mL^−1^ (C_*t*_, 0·625 *μ*g mL^−1^) SCYX-7158 for 24 h. Interestingly, at the next highest concentration studied (C_*u*_, 1·11 *μ*g mL^−1^ equivalent to a C_*t*_, 1·25 *μ*g mL) this target AUC was achieved by 6 h; however, parasites were able to tolerate the higher concentration, for 8 h without impact on survival thereby suggesting treatment time rather than absolute concentration maybe the more important factor in determining efficacy. This observation will be important when designing a dosing regimen for *in vivo* efficacy studies because it implies sustaining exposure above an MIC level (AUC/MIC) maybe more important than achieving a maximum concentration. Further, tolerability in clinical treatment regimens is often limited by the maximum concentration achieved shortly after dosing so the opportunity for lower doses and hence more modest maximal concentrations may improve safety margins and patient compliance.

#### *In vivo* pharmacokinetics and CNS penetration of oxaboroles

Total brain concentration is a helpful first step in assessing CNS penetration relative to MIC to prevent progression of compounds that do not achieve sufficient exposure and would likely fail in Stage 2 efficacy models. A standard *in vivo* protocol would involve dosing the target pre-clinical efficacy species, usually rodents, by intended administration route and then collecting paired blood and tissue samples at selected times post-dose to allow calculation of the tissue-to-plasma (or whole blood) ratio for each sample pair and, more reliably, the ratio based on overall exposure (AUC, area under the concentration *vs* time profile). Total brain exposure is first corrected for the amount of drug in the residual blood using the following parameters: plasma volume, ∼15 *μ*l g^−1^ brain tissue (Vérant *et al.* 2007; Fridén *et al.* 2009), murine microvascular haematocrit, 30% (Vérant *et al.* 2007); density of brain tissue, 1·043 g mL^−1^ (Allen *et al.* 1959), and density of blood 1·046 g mL^−1^ at 30% haematocrit (Friedman, 1959).

AN2920, the original lead compound, achieved moderate plasma exposure maintaining concentrations above its MIC to approximately 7 h post dose, consistent with Stage 1 efficacy (Fig. 4); but CNS exposure above MIC was only achieved for 1 h. AN3520 was selected based on improved plasma exposure and potency and modestly extended CNS exposure; but concentrations in CNS were not consistent with likely efficacy in the CNS model of the disease even if dosed twice daily. In contrast, SCYX-6759 and SCYX-7158 maintained total CNS concentrations above their MIC for approximately 10 and 20 h, respectively, potentially consistent with twice or once daily dosing in a Stage 2 model. Both compounds were progressed to dose range-finding studies in acute and chronic murine models of HAT (Nare *et al.* 2010; Jacobs *et al.* 2011). PK–PD models based on the unbound brain concentrations of SCYX-7158 *vs* cure rate in the chronic HAT model are described below.

### Tier 3: Pharmacokinetics–pharmacodynamics

#### Pharmacokinetics–pharmacodynamics of SCYX-7158 in the Stage 2 (CNS) murine HAT model

*In vitro* reversibility assays for SCYX-7158 indicated that exposure time and AUC/MIC were important in predicting efficacy. The minimum AUC based on unbound concentration and 24 h exposure for complete and irreversible inhibition of parasite growth was 5·81 *μ*g h mL^−1^. This corresponds to a target average unbound concentration at steady-state (C_uSS(AVE)_) in brain of 0·242 *μ*g mL^−1^ which, by definition, corresponds to the unbound MIC. Tantalizingly, partial trypanocidal activity, 24% reduction in survival relative to untreated control parasites (Fig. 8), was also observed in the reversibility assay at a lower concentration (C_*u*_, 0·123 *μ*g mL^−1^; C_*t*_, 0·313 *μ*g.mL^−1^) after 24 h incubation, thereby suggesting that prolonging exposure may allow for a lower dose.

**Fig. 9.**
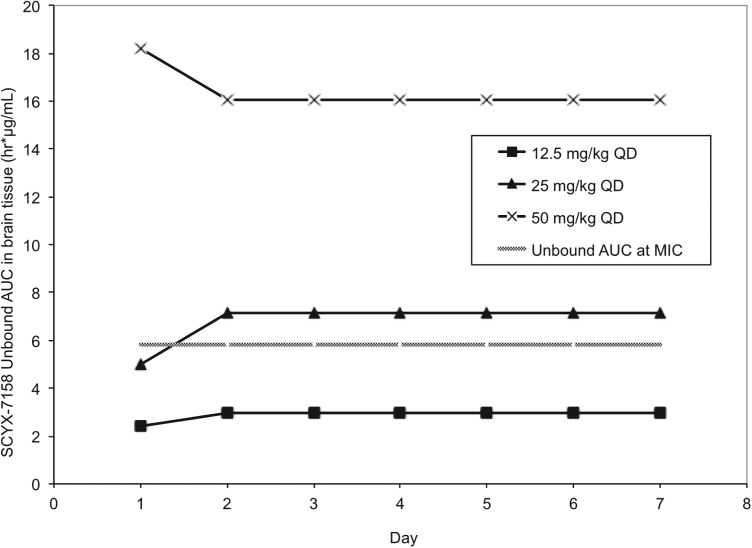
Values for unbound AUC_0–24_ brain tissue collected from uninfected Swiss Webster mice following between 1–7 daily doses of either: 12·5, 25 or 50 mg kg^−1^ SCYX-7158. Values for unbound AUC_0–24_ were calculated using the single (day 1) and steady-state (day 7) exposure data. Non-parametric super-positioning of single dose data indicated steady-state was mostly attained on day 2 so the steady-state data following 7 days of dosing were employed for days 2–7 rather than modelled data for days 2–6. The unbound MIC line represents the lowest concentration that caused irreversible inhibition of parasite growth following 24 h exposure in the *in vitro* reversibility assay.

Pharmacokinetic studies were performed in CD-1 mice to establish AUC_0–24_ following single and 7×once daily oral doses of 6·25, 12·5, 25, 50 and 100 mg kg^−1^ SCYX-7158 (Jacobs *et al.* 2011). Steady-state data were generated in uninfected mice dosed concurrently with those being treated following infection with *T. b. brucei* (TREU 667) for the Stage 2 HAT model. Values for unbound AUC_0–24_ in brain were calculated using the single and steady-state exposure data (non-parametric super-positioning indicated steady-state was mostly attained on day 2 so day 7 data were employed for days 2–7 rather than modelled data for days 2–6) and compared to the target unbound AUC from the *in vitro* reversibility studies (Fig. 9).

All animals treated with the lowest oral dose (6·25 mg kg^−1^ day^−1^) for 7 days were cleared of haemolymphatic *T. b. brucei* but demonstrated recrudescence of parasitaemia around day 35 after infection. Treatment with 12·5 mg kg^−1^ SCYX-7158 once daily for 7 days achieved an 80% cure rate, and higher doses (⩾25 mg kg^−1^ day^−1^) afforded complete cures. Comparison of unbound AUC_0–24_ in brain tissue (Fig. 9) on study days 1–7 demonstrated that animals receiving 25 mg kg^−1^ approached the reversibility assay MIC_u_ AUC_0–24_ (5·81 *μ*g h mL^−1^) on day 1 (4·98 *μ*g.h mL^−1^) and exceeded it for treatment days 2–7 (7·15 *μ*g.h mL^−1^) suggesting that the reversibility data were predictive of efficacy. Interestingly, the unbound AUC_0–24_ for the animals receiving 12·5 mg kg^−1^ SCYX-7158 was approximately 50% of the target MIC_u_ AUC (day 1, 2·41 *μ*g.h mL^−1^ and day 2–7, 2·98 *μ*g.h mL^−1^) yet yielded 80% cures, thus suggesting prolonged exposure at lower AUC can achieve cures.

Examination of the unbound concentration *vs* time curves (not shown) for the animals receiving 12·5 mg kg^−1^ SCYX-7158 indicated that unbound plasma concentrations in brain tissue exceeded the MIC_u_ (0·242 *μ*g mL^−1^) and IC_50u_ (0·082 *μ*g mL^−1^) for only 4 and 10 h, respectively on each day. *In vitro* reversibility data indicated that both exposure time and AUC are important for efficacy and thus the administration of the 12·5 mg kg^−1^ as a divided dose (2×6·25 mg kg^−1^) on a Q12 h regimen may improve efficacy beyond 80% by maintaining exposure for longer, albeit with lower values for Cmax. This hypothesis could be also evaluated *in vitro* by extending exposure times in the reversibility assay.

The need for continued exposure was further indicated in a separate murine Stage 2 HAT study where infected mice were treated with 50 mg kg^−1^ SCYX-7158 for either 1, 3, 5 or 7 days. As expected, a single oral 50 mg kg^−1^ dose achieved an unbound AUC_0–24_ (18·2 *μ*g h mL^−1^) that markedly exceeded the *in vitro* MIC_u_ AUC (5·8 *μ*g h mL^−1^) allowing animals to achieve a potentially therapeutic CNS exposure after a single treatment; however, 100% cure rates were only achieved after ⩾3 days of dosing. No animals were cured after a single dose, thereby suggesting *in vivo* exposure beyond 24 h is required.

## CONCLUSIONS

In conclusion, we have successfully utilized a data-driven strategy based on target tissue disposition, complemented with *in vitro* susceptibility, time-kill and reversibility assays to deliver SCYX-7158 as a potential oral treatment for stage 2 (CNS) HAT. The *in vitro* reversibility assay was employed to correlate exposure based on unbound concentrations with activity, and to identify that exposure time with AUC/MIC were the PK–PD measures that would best predict efficacy in a murine model of chronic (CNS) HAT. Finally, pharmacokinetic studies in uninfected mice dosed concurrently with those infected with *T. b. brucei* (TREU 667) demonstrated that 100% cure rates are achieved when the unbound AUC_0–24_ in brain tissue meets or exceeds the MIC_u_ AUC determined in the *in vitro* reversibility assay. Both the *in vitro* reversibility assay and the murine model suggest that cures maybe achieved at a lower exposure providing exposure is maintained for extended periods. Additional studies to explore this possibility further are planned and will be reported in due course.
